# Reply to Schimmelbusch, K.J.; Collison, F.T. Comment on “Rosa et al. Optic Nerve Drusen Evaluation: A Comparison between Ultrasound and OCT. *J. Clin. Med.* 2022, *11*, 3715”

**DOI:** 10.3390/jcm12175697

**Published:** 2023-09-01

**Authors:** Nicola Rosa, Maddalena De Bernardo, Giulia Abbinante, Gianluca Vecchio, Luigi Capasso, Ferdinando Cione

**Affiliations:** 1 Department of Medicine, Surgery and Dentistry, Scuola Medica Salernitana, University of Salerno, Via S. Allende 43, 84081 Baronissi, Italy; nrosa@unisa.it (N.R.); nandocione1993@gmail.com (F.C.); 2 Corneal Transplant Unit, ASL Napoli 1, 80100 Naples, Italy

We appreciate the comments of Schimmelbusch et al. [1] on our article [2].

**Figure 1:**
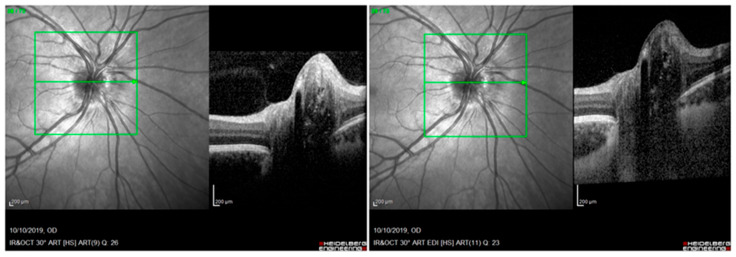
Optical coherence tomography (OCT): optic disc drusen (ODD), visible as hypo-reflective structures with a total or partial hyperreflective margin, as described by the ODD studies consortium. On the left: ODD without enhanced depth imaging (EDI) acquisition modality; on the right: ODD using EDI acquisition modality. Both images are acquired at the same level of the optic disc (green arrow inside the green box).

**Figure 2:**
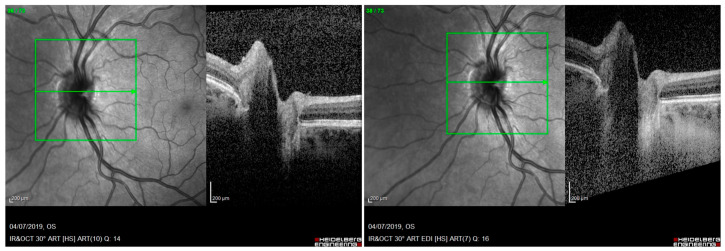
Optical coherence tomography (OCT): hyperreflective peripapillary structure similar to an ovoid mass peripapillary hyperreflective ovoid mass structure (PHOMS). On the left: PHOMS without using enhanced depth imaging (EDI) acquisition modality; on the right: PHOMS using EDI acquisition modality. Both images are acquired at the same level of the optic disc (green arrow inside the green box).

(1) We apologize for the misunderstanding. Figures 3 and 4 do not represent enhanced depth imaging (EDI) OCT acquisition of optic disc drusen (ODD) and peripapillary hyperreflective ovoid mass-like structures (PHOMSs). The purpose of those images was only to represent how they appear in OCT scans, and they were not used for testing OCT sensitivity to detect ODDs and PHOMSs. In our study we acquired both EDI and non-EDI images to recognize both ODDs and PHOMSs, as shown in Figure 1 and Figure 2, but only EDI images were analyzed according to ODDs consortium protocols [3]. Following the example of other similar studies [4], we decided to publish non-EDI figures, but we forgot to highlight that the selected images were not in EDI. However, to avoid misunderstandings, a detailed description of the acquiring protocols of OCT images could be useful.
(2) The authors confuted the role of ultrasound (US) in ODD detection. To support their opinion, they cited a study that reported a low sensitivity rate in ODD detection with US [5]. Actually, according to the cited article, the low sensitivity rate was present only when the exam was performed through the lens (axial scan). On the contrary, when the lens was avoided (parabulbar scan), the sensitivity rate was 100%, and parabulbar US scan was chosen as the exam of choice in ODD detection [2,5]. In addition, other contemporary similar studies that analyzed OCT sensitivity in ODD detection chose US as the baseline exam in ODD recognition. Ref. [6] Malmqvist et al. also reported that the absence of ODD on OCT does not entirely rule out the diagnosis; other modalities are needed, among which include B-scan US [4]. For this reason, to detect ODD in our study, a parabulbar exam with US was performed, as happens whenever other kinds of optic nerve diseases are suspected, avoiding axial scans [7,8].
(3) We agree with the authors that PHOMSs can be found in several conditions and should not be considered as ODD markers, but, as PHOMSs are considered possible markers of ODD by several authors [3,9,10], we decided to perform a US-OCT comparison, both including and excluding PHOMSs, in attempt to achieve the maximum sensitivity value in the OCT detection of ODDs. Unfortunately, in each scenario, OCT did not achieve US standards in ODD evaluation. Therefore, considering the debate in the literature around the role of PHOMSs in ODD detection [3,9,10], we demonstrated that OCT is not a valid alternative to the ODD detection method even if PHOMSs are included. The higher US sensitivity could be related to the presence of calcium deposits that are very well detected with US, but not with OCT.

We are confident that the results of our study are reliable and well documented. We thank the authors again for their comment on our article because they provided us with the opportunity to better explain some of the aspects of our work. Obviously, a multimodal approach including US, Fundus autofluorescence (FAF), and EDI-OCT could improve the strength of the diagnosis of optic nerve head drusen.
